# Asteraceae* Artemisia campestris* and* Artemisia herba-alba* Essential Oils Trigger Apoptosis and Cell Cycle Arrest in* Leishmania infantum* Promastigotes

**DOI:** 10.1155/2016/9147096

**Published:** 2016-10-11

**Authors:** Zohra Aloui, Chokri Messaoud, Meriam Haoues, Noura Neffati, Imen Bassoumi Jamoussi, Khadija Essafi-Benkhadir, Mohamed Boussaid, Ikram Guizani, Habib Karoui

**Affiliations:** ^1^Laboratoire d'Epidémiologie Moléculaire et Pathologie Expérimentale Appliquée aux Maladies Infectieuses LR11IPT04, Université de Tunis El Manar, Institut Pasteur de Tunis, 13 Place Pasteur, BP 74, 1002 Tunis, Tunisia; ^2^Unité Ressources Phytogénétiques et Biotechnologie Végétale, INSAT, BP 676, 1080 Tunis, Tunisia; ^3^Laboratoire de Recherche sur la Transmission, le Contrôle et l'Immunobiologie des Infections LR11IPT02, Université de Tunis El Manar, Institut Pasteur de Tunis, 13 Place Pasteur, BP 74, 1002 Tunis, Tunisia

## Abstract

We report the chemical composition and anti-*Leishmania* and antioxidant activity of* Artemisia campestris* L. and* Artemisia herba*-*alba* Asso. essential oils (EOs). Our results showed that these extracts exhibit different antioxidant activities according to the used assay. The radical scavenging effects determined by DPPH assay were of IC_50_ = 3.3 mg/mL and IC_50_ = 9.1 mg/mL for* Artemisia campestris* and* Artemisia herba*-*alba* essential oils, respectively. However, antioxidant effects of both essential oils, determined by ferric-reducing antioxidant power (FRAP) assay, were in the same range (2.3 and 2.97 mg eq EDTA/g EO, resp.), while the* Artemisia herba*-*alba* essential oil showed highest chelating activity of Fe^2+^ ions (27.48 mM Fe^2+^). Interestingly, we showed that both EOs possess dose-dependent activity against* Leishmania infantum* promastigotes with IC_50_ values of 68 *μ*g/mL and 44 *μ*g/mL for* A. herba*-*alba* and* A. campestris*, respectively. We reported, for the first time, that antileishmanial activity of both EOs was mediated by cell apoptosis induction and cell cycle arrest at the sub-G0/G1 phase. All our results showed that EOs from* A. herba*-*alba* and* A. campestris* plants are promising candidates as anti-*Leishmania* medicinal products.

## 1. Introduction

Leishmaniases consist of complex of infectious diseases caused by protozoan parasites belonging to the genus* Leishmania.* Leishmaniases are widespread, reported in at least 98 countries [1], and considered as major health problems [2]. Leishmaniases could occur as a more or less severe cutaneous disease which affects annually 1.5 million people worldwide. It occurs also as a systemic visceral pathology, which affects annually 500,000 people [3] and could be fatal if left untreated. This results in a high morbidity and in huge financial and quality-of-life losses.

In spite of this epidemiological situation, there is no available vaccine until now. However, many leishmaniasis candidate vaccines (recombinant protein-based and DNA-based vaccines) are at various stages of preclinical and clinical development [4]. Among them, Leish-111 is currently under phase II clinical trials [5]. The first-line chemotherapy of leishmaniasis is mainly based on antimony. However, this drug is toxic and could cause serious side effects [6]. Second-line chemotherapy (such as amphotericin B and miltefosine) is also toxic and requires close clinical control. Therefore, the development of new accessible drugs, which can improve current therapies without undue toxicity, constitutes a research priority for the treatment of leishmaniasis.

Naturally occurring plants offer a new source for screening of antileishmanial activities, considering that nowadays many of the bioactive compounds from plants constitute part of active principles of medicaments. Out of over 200,000 plant species on earth [7] only a small number of specimens have been examined against protozoan parasites [7, 8]. Therefore, this resource needs more investigation in order to assess their effectiveness in the fight against parasites.

Crude extracts from Asteraceae plants, of the genus* Artemisia* in particular, have been previously investigated against protozoan parasites. The well-known and potent antimalarial drug, artemisinin, which originates from the Chinese herb* Artemisia annua* L., is currently used as artemisinin combination therapy for treating malaria [9].* Artemisia* is a heterogeneous genus consisting of more than 500 diverse species occurring in the temperate areas throughout the world but mainly in Europe, Asia, and North America [10].

The antileishmanial activities of some extracts from* Artemisia* species have been reported earlier. The essential oil of* Artemisia absinthium* from Ethiopia was active against* L. aethiopica* and* L. donovani* strains [11]. In the same context, in 2014, Monzote and coworkers have described the antileishmanial activity of* Artemisia absinthium* essential oil from Cuba against promastigotes and amastigotes of* L. amazonensis* [12]. A previous report described the effect of the essential oil of* Artemisia herba*-*alba* from Morocco against* L. tropica* and* L. major* [13]. More recently, the leaves and seeds extracts from* Artemisia annua* from India have been shown to exhibit potent antileishmanial effects [14]. Furthermore, the essential oil from the same species was found to be active against* L. donovani* [15].

In Tunisia, the genus* Artemisia* is represented by five species:* A. arborescens* (L.),* A. vulgaris* (L.),* A. atlantica* (Cross et Dur),* A. herba*-*alba* (Asso.), and* A. campestris* (L.) [16–18].* A. herba*-*alba* (A. ha) and* A. campestris* (A. c) have expanded in semiarid to arid bioclimate. Shrubs, growing in arid and deserted areas, are often used in the folk herbal medicine and by the local people for the treatment or prevention of a number of diseases.

Traditionally, A. ha has been used in the treatment of a variety of ailments such as cold, diabetes, and bronchitis. A previous study has shown that the aerial parts of A. ha are free of undue toxicity [19] and exhibit many pharmacological activities. Aqueous and methanol extracts of A. ha shoot from Jordan have interesting antioxidant capacities [20]. Similarly, A. ha Asso. decoction from Tunisia was reported to possess beneficial antioxidant effect in rats [21].

A. c is mainly known for its use to treat digestive disorders. Previous studies reported its antioxidant effect and antiproliferative activity against HT-29 cancerous cells [22]. Aqueous extract of aerial parts of this medicinal herb had shown a hypoglycemic effect in alloxan-induced diabetic mice [23]. Moreover, its hydroethanolic extract has been reported to have anthelmintic [24] and antimicrobial [25] effects. Recently, essential oils from A. ha and A. c have been described to present antileishmanial activities [26]. Notwithstanding, this report has not assessed their mechanisms of action.

Little studies are currently available on the chemistry of the different preparations (harvesting, drying, storage, etc.) of the two plants. The essential oils components of these two species are mainly monoterpenes (hydrocarbons and oxygenated) and sesquiterpenes (hydrocarbons and oxygenated). However, the aqueous and organic extracts contain phenolic compounds: phenols, flavonoids, flavonols, and flavanols. A. ha has been shown previously to contain methylated flavonoids such as Hispidulin and Cirsilineol [27, 28]. More recently, our team has showed that methanolic extract from A. ha was mainly represented by C-glycosyl flavonoids and caffeoylquinic acids [29].

The aim of this study was to investigate chemical compositions, antioxidant properties, and antileishmanial molecular mechanism of essential oils from these two* Artemisia* species. Our work aims at a thorough investigation of* Artemisia* species in order to characterize their genetic, karyotype, and chemotype diversities in addition to their biological effects. The most interesting genotype(s) will be selected for the requirement of therapeutic and industrial use. Herein, antileishmanial activity was assessed against the proliferation of* Leishmania infantum* promastigotes, the main causative agent of visceral leishmaniasis.

## 2. Material and Methods

### 2.1. Reagents

RPMI 1640 medium and fetal bovine serum were from Gibco. Amphotericin B was from Invitrogen Life Technologies. Resazurin salt, propidium iodide, and RNase were from Sigma Aldrich. Annexin V/7-amino-actinomycin D (7-AAD) apoptosis detection kit was obtained from BD Bioscience. LDH cytotoxicity assay kit was from Roche. Fast staining kit (RAL-555) was from RAL Reactifs. All other reagents are of analytical grade from different commercial sources.

### 2.2. Plant Material and Essential Oil Extraction

*A. herba*-*alba* Asso. and* A. campestris* L. samples were collected from the region of Gafsa, Tunisia (latitude 34° 25′N, longitude 8° 47′E, altitude 298 m, subarid bioclimate) on August 2011 at the flowering stage. The plants were identified by Professor Mohamed Boussaid and voucher specimens were deposited in the herbaria of National Institute of Applied Science and Technology of Tunisia for future reference. The aerial parts (stems and leaves) were used to obtain the essential oils. An amount of 150 g of ground material of each species was hydrodistilled in 1 L of water during 3 hours in a clevenger type apparatus. The obtained oil was dried using anhydrous sodium sulphate, centrifuged, weighed, and then stored at 4°C until analyses [29]. The oil yield was calculated in terms of % of its dry weight. Before stimulating parasites, the oils were filtered using 0.22 *μ*m filter syringe.

### 2.3. Essential Oil Composition

The compositions of the essential oils were determined, as described previously [29], by GC/MS with an Agilent Technologies 7890A apparatus equipped with a HP-5MS fused silica column (30 m × 0.25 mm; 0.25 *μ*m film thickness) and split mode injection (split ratio 1 : 50) where the oil samples were diluted in 50% hexane before injection. Mass spectrometry (MS) analysis was performed on an Agilent mass selective detector 5975 C inert MSD. MS quadrupole temperature was 150°C and mass scan ranged from 50 to 550 amu at 70 eV at a velocity of 2.91 scans/s. The temperature program was 60°C for 2 min, and then it was gradually raised to 240°C at 4°C/min; injector temperature was 250°C. The carrier gas was helium (0.8 mL/min). The identification of compounds was based on comparison of their relative retention indices determined in relation to a homologous series of* n*-alkanes (C9–C24). The volatile components of the essential oils were also identified by automated comparison of their mass spectra with those of the NIST08 (National Institute of Standards and Technology) and W8N08 libraries.

### 2.4. Antioxidant Activity

Three tests were used to compare the antioxidant properties of different plant extracts: DPPH (2,2-diphenyl-1-picrylhydrazyl) radical scavenging assay; chelating effect on ferrous ions; and ferric-reducing antioxidant power (FRAP) method. These methods characterize the ability of the tested extracts to scavenge free radicals and/or to complex metal ions driving the oxidation process.

Briefly, the DPPH method consists of adding 50 *μ*L of the sample in a serial dilution to 0.95 mL DPPH solution (60 *μ*M) and the absorbance (OD) was measured at 517 nm after 60 min of incubation [30]. The inhibition percentage was calculated as follows:

(1) $$\begin{array}{c} \text{Inhibition}\%\left(\;IR\;\right)=100\times \left[\;1-\left(\;\frac{{OD}_{\text{sample}}}{{OD}_{\text{control}}}\;\right)\;\right]. \end{array}$$

The concentration providing 50% inhibition (IC_50_) was calculated from the graph of inhibition percentage against sample concentration. Trolox was used as positive control.

For the ferric-reducing power (FRAP) assay, a FRAP reagent solution was freshly prepared by mixing 300 mM acetate buffer, pH 3.6, TPTZ solution (10 mM 2,4,6-tripyridyl-s-triazine (TPTZ) in 40 mM HCl), and 20 mM FeCl_3_·6H_2_O in a ratio of 10v : 1v : 1v [31]. To perform the assay, 0.9 mL of FRAP reagent, 90 *μ*L of distilled water, and 30 *μ*L of each sample (or water in the case of blank tube) were mixed and incubated at 37°C for 30 min. The absorbance was measured at 595 nm. The antioxidant potential of samples was determined from a standard curve plotted using the FeSO_4_·7H_2_O linear regression. The results were corrected for dilution and expressed as mmol of Fe^2‏+^·g^−1^ of essential oil.

The ferrous ion chelating activities of essential oils from the studied plants were measured according to the method described by Messaoud et al., 2012 [30]. Increasing concentrations of essential oils (1 mg/mL; 1.5 mg/mL; and 10 mg/mL) prepared in 0.3 mL methanol were added to 0.3 mL of 0.1 mM FeSO_4_ solution and incubated at room temperature (25–28°C) for 5 min. The mixture was vortexed thoroughly after the initiation of the reaction (with 0.3 mL of 0.25 mM ferrozine) and then incubated for 10 min at room temperature. The ferrous ion chelating activities were determined by the absorbance at 562 nm. The formula chelating effect (%) = [100 × (*A*_*c*_ − *A*_*S*_/*A*_*c*_)] was used to determine the essential oils ability to chelate ferrous ion, where *A*_*c*_ is the absorbance of the control sample (mixture of methanol, iron, and ferrozine) and *A*_*S*_ the absorbance of the tested sample. Results were expressed as IC_50_ (efficient concentration corresponding to 50% ferrous iron chelating). EDTA was used as positive control.

### 2.5. Ethical Statement

All work on mice was compiled under the European council 2010/63/EU directive and with all relevant guidelines and institutional policies.* In vivo* passage of parasites in BALB/c mice received approval from the biomedical ethics committee of Pasteur Institute of Tunis (reference number 2016/09/I/LR11IPT04/V0). The peritoneal macrophage isolation protocol from BALB/c mice received approval from the same committee (reference number 2016/10/I/LR11IPT04/V0).

### 2.6. Parasites Culture

Standard laboratory* L. infantum* parasite strain was used in this study [32]. WHO code attributed to this* L. infantum* strain (MHOM/TN/94/LV49) summarizes host, geographical origin, year of isolation, and laboratory code.

Promastigotes of* L. infantum* were then cultured at 22 ± 2°C in RPMI1640 medium supplemented with 10% heat-inactivated fetal bovine serum (FBS), 20 mM HEPES buffer, penicillin G (50 IU/mL), and streptomycin (50 *μ*g/mL). Parasites were cryopreserved in liquid nitrogen and routinely maintained with* in vivo* passages in female BALB/c mice.

### 2.7. *In Vitro* Evaluation of Antipromastigote Activity

After subculturing, promastigotes in the logarithmic growth phase were seeded in 24-well plates (10^6^ promastigotes/mL) and incubated with increasing concentrations of essential oil (diluted in dimethyl sulfoxide (DMSO)) at 24, 48, and 72 h. EOs were sterilized using a 0.22 *μ*m membrane filter before stimulating parasites. The antileishmanial activity was assessed by measuring cell viability with trypan blue exclusion staining as described by Strober, 2001 [33], and the number of parasites was counted in a Neubauer hemocytometer. Numbers of parasites, in the control wells, grown in the presence of vehicle (0.05% DMSO) were used as maximum values (100%). Amphotericin B, the standard antileishmanial drug, was used as a positive control.

The antileishmanial activity was also assessed by resazurin dye. In this case, promastigotes at log-phase were seeded in sterile 96-well plates (2 × 10^6^ cells/mL) and incubated with increasing concentrations of essential oils. Negative controls correspond to cells (wells) exposed to 0.05% DMSO. After 12 and 60 h incubation with essential oils, resazurin dye was added at each well at a final concentration of 2.5 *μ*g/mL [34, 35] and allowed to incubate for further 12 h. Cell viability was monitored with a fluorometer (*λ*_ex_ 550 nm; *λ*_em_ 590 nm) (VarioSkan reader, Thermofisher Scientific, Vantaa/Finland). The mean percentage viability was calculated as follows:

(2) $$\begin{array}{c} \%\;\;viability=\frac{fluorescence\text{ in treated wells (parasites)}-fluorescence\text{ in blank wells}}{fluorescence\text{ in negative control wells}-fluorescence\text{ in blank wells}}\times 100. \end{array}$$

The inhibitory concentration that decreased the cell growth by 50% (IC_50_) was determined. Positive control wells were incubated with IC_50_ amphotericin B: 0.03 *μ*g/mL in the first assay (trypan blue assay) and 0.06 *μ*g/mL in the second assay (resazurin assay). All the assays were performed in technical triplicate and were repeated three times on different days (biological triplicate). The final concentration of DMSO in the cultures was adjusted to 0.05% (v/v), a concentration that we determined as nontoxic for the parasites.

### 2.8. Determination of Cellular Morphology

To observe changes in cellular morphology, parasites were incubated with the highest EO concentrations tested (0.5 *μ*L/mL) which are equivalent to 220 *μ*g/mL and 460 *μ*g/mL, respectively, for A. c and A. ha essential oils, compared to 0.5 *μ*g/mL amphotericin B. Control and treated parasites were harvested by 1800 rpm (630 g) centrifugation. Aliquots of the suspension were placed on glass slides, sealed, fixed with ethanol, and stained with 10% Giemsa dye solution [36]. Parasites were then observed under 40x and 100x magnifications. To record any changes in cellular morphology, control and treated cells at the different concentration conditions were observed at two different time points (24 and 72 h).

### 2.9. LDH Cytotoxicity Assay

This assay was used to evaluate plasma membrane damage in parasites. The lactate dehydrogenase (LDH) is a stable cytoplasmic enzyme that is rapidly released into the cell-culture supernatant when the plasma membrane is damaged [37]. Parasites (10^6^/mL) were cultured in 24-well plates and stimulated with both essential oils at the highest concentrations tested (0.5 *μ*L/mL), while vehicle- (0.05% DMSO-) treated cells were used as controls. After 24 and 72 h incubation, a 100 *μ*L aliquot of the culture supernatant at each condition and time point was collected and incubated with the substrate mixture from the kit (LDH cytotoxicity kit, Roche) as indicated by the manufacturer. LDH activity is determined in a coupled enzymatic reaction in which the tetrazolium salt INT is reduced to formazan. The quantity of formazan, which is proportional to LDH activity, is determined by measuring absorbance at 492 nm. Assays were repeated three times with technical duplicates.

### 2.10. FACS Analysis of Cell Cycle

Cell cycle analysis by quantification of DNA content was performed by flow cytometry. Parasites (10^6^/mL) were treated with increasing concentrations of both essential oils for 24 and 72 h. At each time point, cells were fixed with 70% chilled ethanol and kept at −20°C until analysis. After washing twice with PBS, cells were treated with DNase-free ribonuclease (20 *μ*g/mL) and stained with 20 *μ*g propidium iodide (PI) for 15 min at room temperature in the dark [37]. Data acquisition was carried out using a FACS flow cytometer and analyzed using CellQuest software.

### 2.11. FACS Analysis for Determination of Phosphatidylserine (PS) Externalization

For the detection of apoptotic or necrotic cell death, the annexin V/7-amino-actinomycin D (7-AAD) apoptosis detection kit (BD Bioscience) was used according to the manufacturer's protocol. After 24 and 72 h incubation with essential oils, promastigotes (10^6^ cells/mL) were washed in phosphate-buffered saline (PBS) and centrifuged at 1800 rpm (630 g) for 10 min. Then, they were stained with annexin V-PE/7-AAD for 15 min in the dark at room temperature [37]. Acquisition was performed using a BD Biosciences flow cytometer. Data were analyzed using the CellQuest software and the percentage of positive cells was determined for each sample.

### 2.12. Cytotoxicity Assay on Mouse Macrophages

Mouse peritoneal macrophages were harvested from 6–8-week-old female BALB/c as described previously [38, 39]. The cells were cultured at 37°C and 5% CO_2_ (6 × 10^5^ cells/mL) in 96-well plates (200 *μ*L/well) for MTT assay and in 24-well plates (1 mL/well) for coloration. Cells were allowed to adhere for 24 h. After washing with PBS to eliminate nonadherent cells, macrophages were stimulated with different concentrations of essential oils (0–240 *μ*g/mL) for 24 h. Cytotoxicity was evaluated by adding MTT (1 mg/mL each well) [37] to the 96-well plates. The supernatant was discarded and the formazan crystals were dissolved with 100 *μ*L DMSO/well. Absorbance was then measured at 540 nm using an ELISA plate reader. The 50% cytotoxicity concentration (CC_50_) on BALB/c peritoneal macrophages was calculated with the GraphPad Prism Software. Cells in 24-well plates were washed with PBS, fixed with methanol, and then stained with Kit RAL 555. The test was carried out in triplicate.

### 2.13. Statistical Analysis

All data are expressed as the means ± standard deviation (SD). The data were analyzed using Student's* t*-test. Changes with *p* values less than 0.05 were considered to be statistically significant and *p* < 0.01 was considered highly significant.

## 3. Results

### 3.1. Essential Oil Yield and Composition

A bright yellow essential oil with an intense woody smell was obtained by hydrodistillation of* Artemisia herba*-*alba* yielded to 0.66% of its dry weight. The density of the oil was 0.938 g/mL. Nineteen compounds, representing 98.13% of the total oil, were identified (Table 1). The major components of this essential oil are camphor (36.82%), 1,8-cineole (13.85%), chrysanthenone (8.80%), *α*-thujone (7.65%), and *β*-thujone (7.21%).

The essential oil from* Artemisia campestris* has a dark color with a yield corresponding to 0.41% of dry weight. The density of the oil was 0.880 g/mL. As shown in Table 1, twenty-four compounds, representing 94.95% of the total oil, were identified. The major components of this oil are *β*-pinene (32.95%), limonene (15.13%), *α*-pinene (12.25%), g-terpinene (7.6%), and *β*-myrcene (5.51%).

### 3.2. Antioxidant Activities

The antioxidant activities of the essential oil obtained from both* Artemisia* species were determined. Our results showed that these extracts exhibit potent antioxidant activities, which vary between the two species (Table 2). The A. c essential oil exhibits a potent radical scavenging effect (IC_50_ = 3.3 mg/mL) compared to that of A. ha (IC_50_ = 9.1 mg/mL). Antioxidant effects of both essential oils, determined by ferric-reducing antioxidant power (FRAP) assay, were in the same range (2.3–2.97 mg eq EDTA/g EO), while the A. ha essential oil showed the highest reducing power (27.48 mM Fe^2+^) (Table 2).

### 3.3. Antileishmanial Activities of the Essential Oils

**Figure 1:**
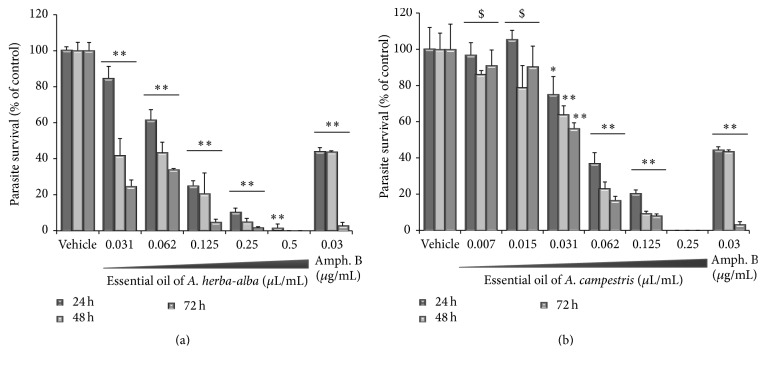
Antileishmanial activity of serially diluted concentrations of essential oil from (a)* Artemisia herba*-*alba* and (b)* Artemisia campestris* against* L. infantum* promastigotes monitored by trypan blue exclusion at 24 (dark grey square), 48 (light grey square), and 72 h (grey square) incubation. Results are reported as the means ± SD of three independent experiments carried out in technical triplicate. Statistical significance was measured by comparing treated groups to control groups (^*∗∗*^ *p* < 0.01; ^*∗*^ *p* < 0.05; ^$^insignificant).

Both essential oils induced loss of viability and growth inhibition on exponentially grown LV49 promastigotes (10^6^ cells/mL). They have shown an interesting dose-dependent decrease in promastigote counts, which is maintained over 72 h incubation (Figure 1). Viability of* L. infantum* was susceptible to EO of A. ha with an IC_50_ of 0.0725 *μ*L/mL, which corresponds to 68 *μ*g/mL and to EO of A. c with an IC_50_ of 0.05 *μ*L/mL (=44 *μ*g/mL), at 24 h exposure. Positive control was assessed by amphotericin B under the same conditions and IC_50_ value against LV49 strain was 0.03 *μ*g/mL, which is similar to the ones reported in the literature [40].

**Figure 2:**
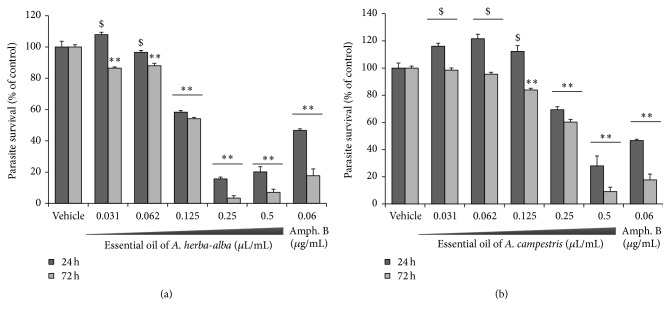
Antileishmanial activity of serially diluted concentrations of essential oil from (a)* Artemisia herba*-*alba* and (b)* Artemisia campestris* against* L. infantum* promastigotes. The activity was determined with resazurin test and monitored after 24 h (dark grey square) and 72 h (grey square) incubation. Results are reported as the means ± SD of three independent experiments carried out in technical triplicate and statistical significance was measured by comparing treated groups to control groups (^*∗∗*^ *p* < 0.01; ^$^insignificant).

The antileishmanial activities of both EO were further showed and quantified using the resazurin dye assay in similar experiments testing the effects on parasites plated at a concentration of 2 × 10^6^/mL (Figure 2). This assay measures the reduction of the cell-permeable dye resazurin into fluorescent resorufin by intracellular enzymes. Treatment of cultured* L. infantum* promastigotes by both essential oils resulted in dose- and time-dependent inhibition of promastigote metabolic activity with IC_50_ value of 0.156 *μ*L/mL (Figure 2(a)) and 0.375 *μ*L/mL (Figure 2(b)) for A. ha and A. c, respectively, at 24 h exposure. Viability of* L. infantum* was susceptible to amphotericin B with IC_50_ = 0.06 *μ*g/mL at the same incubation time.

### 3.4. Effects on Cellular Morphology

**Figure 3:**
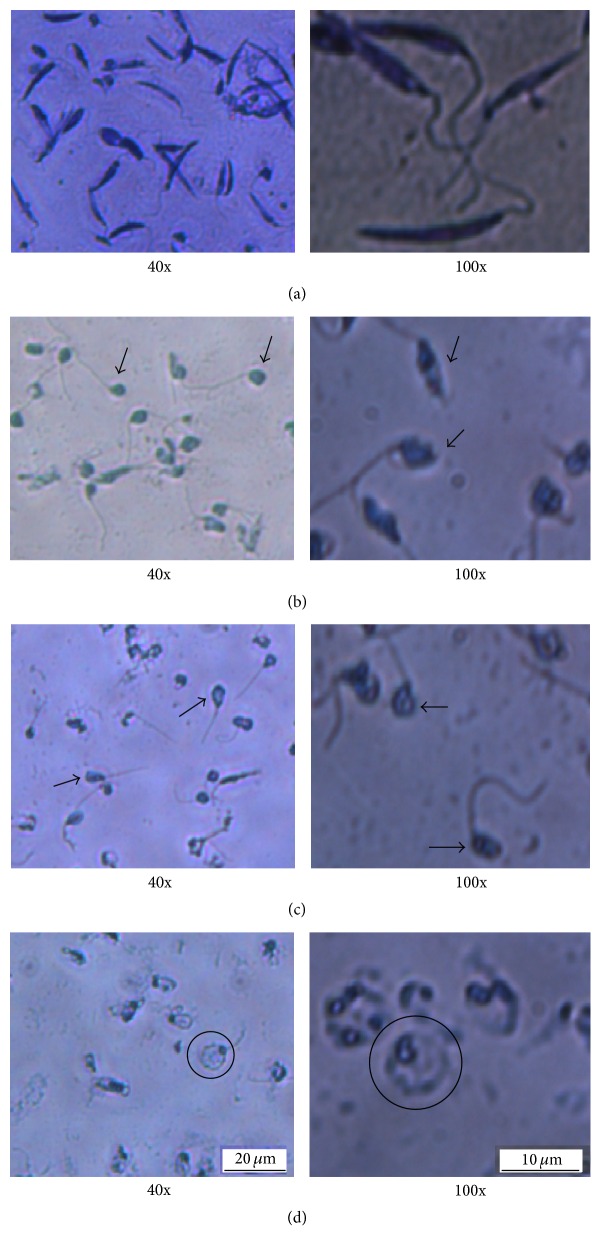
Morphological effect of the essential oils on* L. infantum* promastigotes. The figure illustrates the Giemsa staining of* L. infantum* promastigotes with no treatment (a) or after treatment with 0.5 *μ*L/mL EO of* A. herba*-*alba* (b), 0.5 *μ*L/mL EO of* A. campestris* (c), or 0.5 *μ*g/mL amphotericin B (d). The slides were observed at 40x and 100x magnifications. Untreated cells show ordinary elongated cell bodies while those stimulated with EO present remarkable shrinkage of the body. Treatment with amphotericin B results in swollen promastigote bodies. The arrows point to shrunken promastigote bodies while the circles highlight the swollen promastigote bodies.

Microscopic observation of parasites in presence of 0.05% DMSO showed ordinary elongated cell bodies (Figure 3(a)). However, parasites treated with both EOs showed a different shape with a well-defined flagellum but a shrunken body (Figures 3(b) and 3(c)), which could be due to organelle disruptions and/or chromatin condensation in the nucleus [41]. However, parasites treated with amphotericin B showed round swollen bodies (Figure 3(d)). Herein, optical microscopy did not allow us to conclude about cell membrane integrity.

### 3.5. Effect on Cell Membrane by LDH Release Assessment

**Figure 4:**
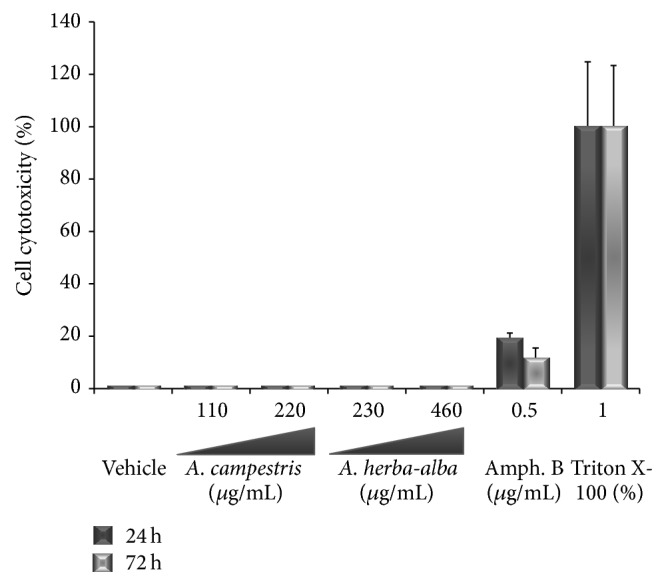
Essential oils of* Artemisia herba*-*alba* and* Artemisia campestris* show little to no cytotoxic effect. Cell lysis was evaluated on the basis of lactate dehydrogenase (LDH) activity released from the cytosol of damaged cells into the culture supernatant. Parasites exposed to essential oils from* Artemisia herba*-*alba* (230 and 460 *μ*g/mL) and from* Artemisia campestris* (110 and 220 *μ*g/mL) showed very low level of cytolysis and amphotericin B (0.5 *μ*g/mL). Cells exposed to 1% Triton X-100 were completely lysed.

The levels of the lactate dehydrogenase (LDH) in the supernatants of both essential oil-treated parasites after 24 and 72 h treatment, as quantified by salt reduction, were comparable to the negative control. Thus, the LDH enzyme was not released in the culture medium even at the highest concentrations tested (0.5 *μ*L/mL), compared with the spontaneous release from negative control wells and from positive control wells treated with Triton X-100 (Figure 4), allowing us to conclude that the inhibitory effect of EOs on* Leishmania* did not trigger cytolysis of* L. infantum* promastigotes even after prolonged incubation.

### 3.6. Effect of Essential Oils on* L. infantum* Promastigotes Cell Cycle

**Figure 5:**
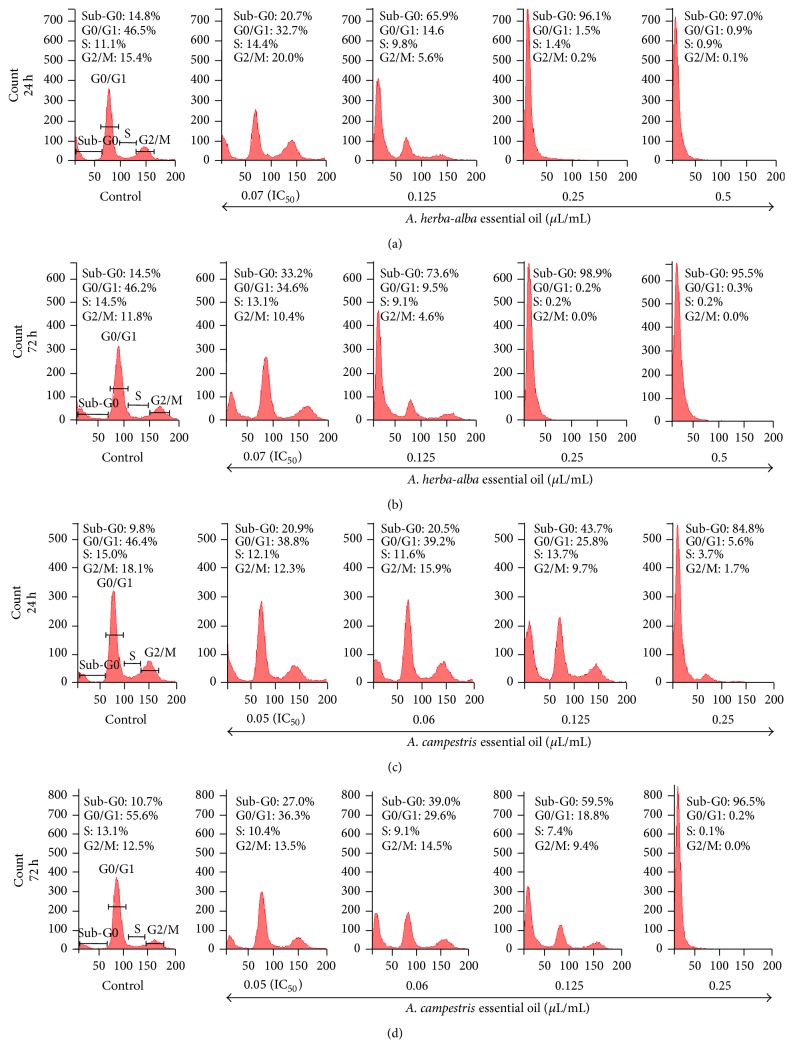
Effect of the essential oils on the* Leishmania* cell cycle. The figure illustrates the cell cycle analysis of promastigotes following treatment with increasing amounts of* Artemisia herba*-*alba* (a, b) and* Artemisia campestris* essential oils (c, d) and staining with propidium iodide (PI). Both essential oils induce cell cycle arrest in* L. infantum* promastigotes at sub-G0 phase as evaluated after 24 h and 72 h treatment.

In an attempt to understand the growth reduction observed after essential oil treatments, we performed a flow cytometry analysis after cell permeabilization and labeling with PI to quantify the DNA content. Thus, the amount of DNA is correlated with the amount of bound dye and fluorescence intensity. In promastigotes incubated with increasing concentrations of A. ha EO for 24 h, the proportion of cells in the sub-G0/G1 phase increased among doses ranging from 20.7% to 97.0% compared with 14.8% of negative control (Figure 5(a)). The same effect was observed in A. c treated promastigotes (Figure 5(c)). Following 72 h of treatment, the proportion of cells in the sub-G0/G1 phase increased further compared to 10.7% of negative control (Figures 5(b) and 5(d)). This increase in sub-G0/G1 phase was accompanied by a decrease in the number of cells in both the S and G2/M phases compared with vehicle-treated cells (Figure 5). Taken together, the increased proportion of cells in the sub-G0/G1 phase indicated DNA degradation in promastigotes and suggested apoptotic-like changes.

### 3.7. Apoptotic Effect of the Essential Oils

**Figure 6:**
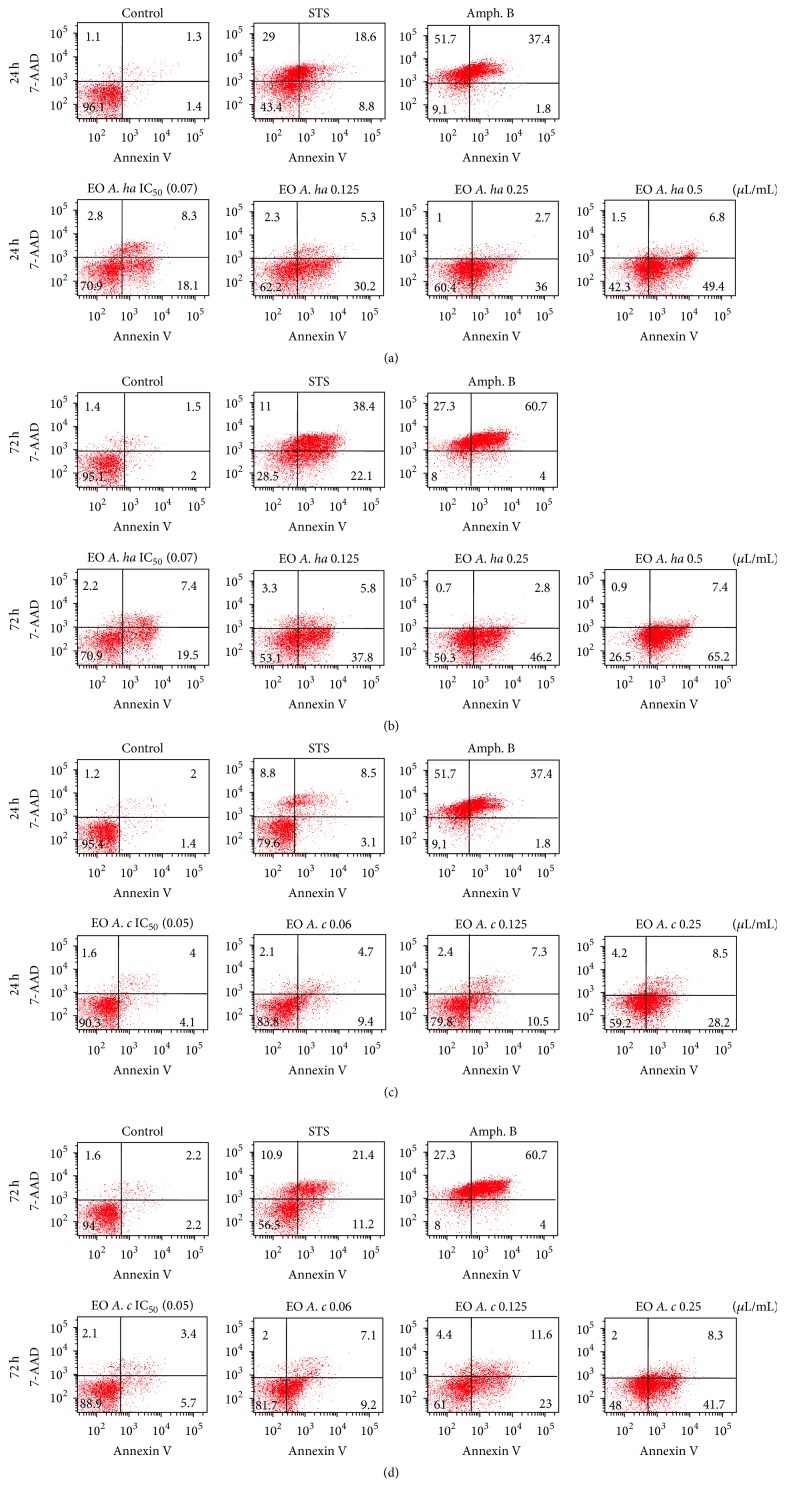
Apoptotic effect of essential oils of* Artemisia herba*-*alba* and* Artemisia campestris*. Promastigotes were analyzed by flow cytometry after treatment with increasing amounts of* Artemisia herba*-*alba* (a, b) and* Artemisia campestris* essential oils (c, d) followed by a double staining annexin V/7-AAD. In control experiments, the parasites were incubated with 0.05% DMSO (vehicle), 1 *μ*M staurosporine (positive control), or 0.5 *μ*g/mL amphotericin B (leishmanicidal reference drug). Both essential oils trigger apoptosis in* L. infantum* promastigotes in a dose- and time-dependent manner.

We then performed a further FACS analysis with double annexin V/7-AAD staining to differentiate between living, apoptotic, and necrotic cells. Staurosporine at 1 *μ*M was used as a positive control as it classically used to induce apoptosis in various eukaryotic cells. Amphotericin B at 0.5 *μ*g/mL was also used as a standard for a leishmanicidal activity (Figure 6).

With the EOs, our results showed that the percent of annexin-positive cells increased according to the doses and the time of incubation. After 24 h treatment with A. ha essential oil within concentrations ranging from 0.07 to 0.5 *μ*L/mL, the percent of annexin-positive cells varied from 18.1% to 49.4%, respectively (Figure 6(a)). These values increased up to 19.5% and 65.2% with the 72 h treatment (Figure 6(b)). In the vehicle (0.05% DMSO) controls annexin-positive parasites did not exceed 2% in both time points. The A. ha EO-treated parasites clearly expressed apoptotic profiles, while the amphotericin B treated parasites stained positive for 7-AAD and rather had a necrotic profile.

Apoptosis was also observed for the A. c EO as quantified by annexin-positive cells; the percent values of annexin-stained cells ranged from 9.2% to 28.2% after 24 h (Figure 6(d)) and from 9.4% to 41.7% after a 72 h treatment (Figure 6(d)), in a dose-dependent manner, while both EOs stained cells remained negative for 7-AAD even after 72 h incubation, confirming that the cell membranes remained intact.

### 3.8. Cytotoxicity against* Leishmania* Target Cells

**Figure 7:**
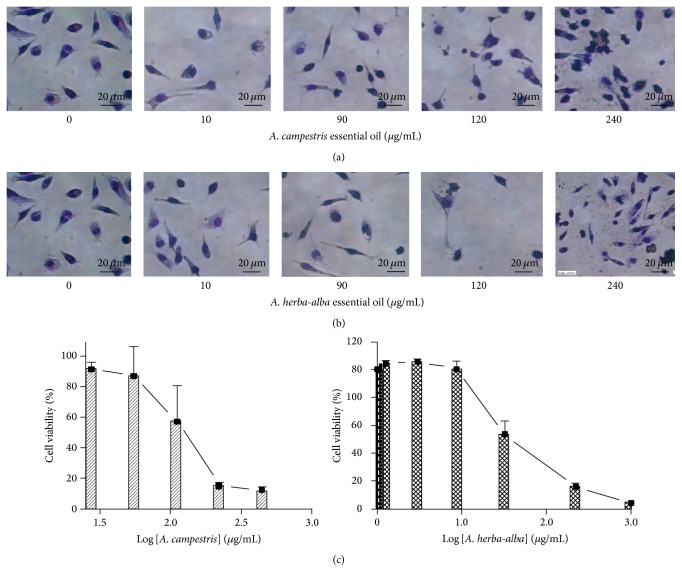
*In vitro* cytotoxicity of* Artemisia campestris* and* Artemisia herba*-*alba* essential oil against BALB/c peritoneal macrophages tested for 24 h. Light microscopy of macrophages treated with gradual concentration (0–240 *μ*g/mL) of (a) A. c essential oil and (b) A. ha essential oil. (c) Viability of peritoneal macrophages treated with the same range (0–240 *μ*g/mL) for CC_50_ determination. Data represents mean ± SD of three independent experiments conducted in triplicate.

Because macrophages are target cells of the infection by* Leishmania*, they have been used to assess the toxicity of both essential oils. Morphology of peritoneal macrophages treated with increasing concentration (0–240 *μ*g/mL) of both essential oils was monitored on cell monolayer stained with Giemsa solution (Kit RAL 555) (Figures 7(a) and 7(b)). Our result from MTT test has shown that CC_50_ for A. c was 124.4 *μ*g/mL and for A. ha was 160 *μ*g/mL (Figure 7(c)). This data gives a selectivity index (SI) of 2.82 and 2.35, respectively, for both essential oils.

## 4. Discussion

In the present study, we investigated the chemical composition, antioxidant, and antileishmanial activities as well as the mechanism of action of essential oils from two* Artemisia* species growing in Tunisia,* Artemisia herba*-*alba* and* Artemisia campestris*.

The essential oil of A. ha collected from a subarid region (Gafsa) comprises 85.79% oxygenated monoterpenes with camphor as the major compound (36.82%). However, differences according to ecogeographical conditions were revealed. Previous study derived with 18 samples of A. ha collected from areas in diverse arid domains of southern Tunisia [42] has showed that 12 oil samples contain 57% oxygenated monoterpenes of the total oils. For these monoterpene-rich samples, only two have got the camphor as the major compound [42]. The other samples had a high proportion of borneol and thujone [42]. Recently, we studied the essential oil of an A. ha sample from Kairouan (Central Tunisia, upper arid bioclimate) and we found *β*-thujone (41.9%) as the major compound [29]. In other countries like Jordan, oxygen-containing monoterpenes represent also the main compounds of the oil [43]. Monoterpene-rich A. h essential oils were also described in Morocco with a defined cineole-camphor chemotype [44].

Conversely, A. c essential oil was characterized by a high content of monoterpene hydrocarbons (87%) with *β*-pinene as major compound (32.95%). The chemical compositions of essential oils from this plant had been investigated by many researchers in Tunisia [22, 45]. The essential oil of a sample collected in the area of Ben Gardane (South-east Tunisia, arid bioclimate) has shown also the *β*-pinene in a high rate of 41.0% [45]. Another specimen from South-east Tunisia (Beni Khedache, mountainous region) was also shown to have monoterpene hydrocarbons as major components with 34.2%  *β*-pinene [22]. However, more recently, Essid and coworkers, 2015, reported a chemical composition of A. c sample collected from the north of Tunisia with *α*-pinene as major component (24%). The intraspecific differences in chemical compositions could be attributed to several factors, including genetic diversity, climatic conditions, geographical locations, harvesting time, and phenological stage [46, 47].

The hydrodistilled essential oils from both species of* Artemisia* showed* in vitro* antioxidant activities that characterize their ability to protect against free radicals involved in many cellular alterations process. A. c essential oil exhibited the highest DPPH scavenging capacity with IC_50_ value of 3.3 mg/mL compared to an IC_50_ = 9.1 mg/mL for A. ha essential oil. Our previous work with a A. ha essential oil sample from the region of Kairouan (Tunisia) showed a slightly higher IC_50_ of 5.03 mg/mL [29] while a sample of A. c essential oil from Beni Khedache (South Tunisia) was shown to be approximately at least tenfold lower in radical scavenging with IC_50_ value of 94.5 mg/mL [22]. Both essential oils showed potent and comparable ion chelating capacities, 2.97 and 2.30 mg EDTA/g EO for A. c and A. ha essential oils, respectively. These values are in the same range of the ion chelating capacity of A. ha essential oil from Kairouan [29]. The ferric-reducing power of A. ha was slightly higher than that of A. c essential oil. Even if a slight difference was observed according to the used assay (DPPH or FRAP) between the two species of essential oils, both could constitute potent antioxidant extracts.

Essential oils from a wide range of* Artemisia* species and some of their components have been shown to possess ethno- and pharmacological properties, particularly against infectious diseases [48]. Herein, we investigate the potential antileishmanial effects of* Artemisia* species growing in South Tunisia. Indeed, essential oils of* Artemisia herba*-*alba* and* Artemisia campestris* proved to be effective against viability and proliferation of promastigotes of* L. infantum*, a parasite responsible for visceral leishmaniasis cases in Tunisia and many other countries worldwide. The inhibitory effects of both EOs towards* L. infantum* were dose- and time-dependent. Camphor-rich (36.82%) EO from A. ha had an IC_50_ of 68 *μ*g/mL while the *β*-pinene-rich (32.95%) EO from A. c had an IC_50_ of 44 *μ*g/mL. The antileishmanial activities of essential oils from other* Artemisia* species were also described.* A. absinthium* EO from Cuba (contains 36.7% trans-sabinyl acetate) was able to kill* L. amazonensis* promastigotes with 50% inhibitory concentration of 14.4 *μ*g/mL [12]. The EO from leaves of* A. annua*, which contain 52.06% camphor, was also potent against* L. donovani* with IC_50_ value of 14.63 *μ*g/mL [15]. Camphor-rich (27.4%) essential oil from* A. absinthium* from Ethiopia has been reported to exhibit activity against the promastigote forms (MIC = 0.1565 *μ*L/mL) of both* L. aethiopica* and* L. donovani* strains [11]. On the other side,* A. abyssinica* from Ethiopia that had been active also against* L. donovani* and* L. aethiopica* (MIC values of 312.5 nL/mL and 76.5 nL/mL, resp.) was mainly rich in yomogi alcohol (38.47%) and artemisyl acetate (24.88%) but possesses camphor as a trace (0.68%) [49]. In a previous work, a preliminary study described the antileishmanial activity of A. ha EO from Morocco with an IC_50_ of 2 *μ*g/mL against* L. major* [13]. To our best knowledge, little studies up to now described leishmanicidal activity of A. c essential oil although it is endowed with interesting bioactivities. While preparing this report, another group have published their studies on antileishmanial potential of some essential oils from medicinal plants in northern Tunisia including* Artemisia campestris* [26] which exhibit an IC_50_ = 3.24 *μ*g/mL against* L. infantum* at 72 h exposure. The difference in time posttreatment can explain the differences in the IC_50_ values between our results and those reported by Essid et al., 2015. Furthermore, it is well documented that EOs chemical composition was subjected to intraspecific variations due to several factors including geographical locations, climatic conditions, and seasonal variations. In our work, plant samples were collected in August from Gafsa located in the south of Tunisia and this region is characterized by subarid bioclimate. However, in the work conducted by Essid et al., 2015, the samples were collected from the north of Tunisia which is characterized by more fresh and more humid climate (upper-humid bioclimatic stage). The major component of A. c EO, *α*-pinene, exhibits also a leishmanicidal effect with IC_50_ = 17.6 *μ*g/mL [26]. In our study, we found that A. c essential oil contains *α*-pinene at only 12.25% but a trace of it (0.26%) was observed in A. ha essential oil. These observations clearly demonstrate that *α*-pinene is not responsible for the whole activity of the essential oils. Although the major components of EOs, generally, determine their biological properties, components in trace amounts can also determine this activity and contribute to its enhancement. Taken together, our observations suggest that there is not one compound that determines the leishmanicidal activity. Indeed, we have two distinct compositions of two essential oils but the same mechanisms of action. Therefore, the inhibitory effects of the essential oils on* Leishmania* growth may be due to synergy effect between the various components of the oil. Although the antileishmanial activity is conducted* in vitro* against promastigote forms, antiamastigote activity is necessary as these are the forms that reside within the macrophages and spread the infection in the host. We have promising results that those safe doses of* Artemisia* essential oils possess antiamastigote activity (Supplementary Materials available online at http://dx.doi.org/10.1155/2016/9147096). Therefore, it would be relevant for the next step to identify the best combination of compounds responsible for the antiamastigotes and antipromastigotes activities. Antioxidant activity of EO may be beneficial for the antileishmanial activity of the plant metabolites. It was reported previously by Zahir et al., 2015 [50], that induced decrease of reactive oxygen species level by* Euphorbia* plant extract is implicated in the death mechanism of* Leishmania* parasites and could be responsible for the caspase-independent shift from apoptosis to massive necrosis. Furthermore, studies done previously by our team [51] have shown that increase in reactive oxygen species induced by* Leishmania* Eukaryotic Initiation Factor (LeIF) in murine macrophages is implicated in the enhancing resistance of macrophages J774 to* Leishmania* infection.

In our study, remarkable morphological changes of the parasites were observed (like shrinkage of the body) in presence of both EOs that reminded morphological features of mammalian apoptosis. LDH cytotoxicity assay and FACS analysis after annexin V and 7-AAD labeling confirmed that essential oils from both species killed the parasites by triggering an apoptosis mechanism rather than by necrosis. PS externalization from the inner to the outer surface of* L. infantum* plasma membrane was increased in response to gradual concentrations of both essential oils, as evidenced by increased annexin V binding. A similar phenomenon was previously observed for several antileishmanial drugs including miltefosine [52], the current oral treatment of visceral leishmaniasis, and* Aloe vera* leaf exudate [53] and* A. annua* leaf EO [15]. Interestingly, amphotericin B, the drug we used in this work as a reference leishmanicidal, induced necrosis in promastigotes. In fact, the major percentage of cells exposed to this drug were stained with 7-AAD suggesting a membrane damage. This is in agreement with evidence that amphotericin B kills* Leishmania* parasites by forming aqueous pores permeable to small cations and anions, which can lead to osmotic lysis [54]. The nuclear material during programmed cell death was further analyzed by examination of cell cycle. In fact, cell analysis with PI showed a dose-dependent increase in the sub-G0/G1 phase of treated promastigotes (Figure 5). The sub-G0/G1 enhanced peak indicates DNA fragmentation. Some other natural or synthetic molecules with antileishmanial activities have been reported to trigger a similar effect such as artemisinin [55], oxoallobetulin [56],* Artemisia annua* leaf essential oils [15], and withanolides from the leaves of* Withania somnifera* [57].

Biomembranes are the first target of an antiparasitic drug. Because parasites share most molecular and biochemical features with their eukaryotic hosts, it is therefore necessary to assess the effects of the EOs on* Leishmania* host target cells. When examined on murine peritoneal macrophages, the EOs show low cytotoxic concentration with CC_50_ values of 124.4 and 160 *μ*g/mL, respectively, for A. c and A. ha EOs (Figure 7(c)). This result was considered relevant as the cytotoxicity falls within the generic hit selection criteria to antileishmanial compounds published by the DNDi and Pan-Asian Screening Network, 2009 [58]; a compound with CC_50_ ≥ 90 *μ*g/mL was classified as nontoxic. In their previous study, Essid et al., 2015, have reported CC_50_ values of 80.6 *μ*g/mL and 8.8 *μ*g/mL, respectively, for A. c and A. ha EOs. These differences may be due to the whole composition differences between the EOs. We should underline also that the differences may be due to different cell lines and cell densities used to carry on cytotoxicity.

## 5. Conclusion

In the present study, we reported chemical compositions and antioxidant and antileishmanial activities of two Asteraceae plants growing in South Tunisia. Camphor-rich essential oil from* Artemisia herba*-*alba* and *β*-pinene-rich essential oil from* Artemisia campestris* exhibited both potent inhibitory activity against* L. infantum* promastigotes in a dose- and time-dependent manner. Both essential oils trigger programmed cell death in* L. infantum* promastigotes, as evidenced by PS externalization and cell cycle arrest in sub-G0/G1 phase, while having distinct compositions. This is the first study to our knowledge that describes the mode of action of these EOs on* Leishmania* parasites. Further studies are required to identify the bioactive compound(s) of* Artemisia* species with a view to investigate them as potential alternative herbal preparations to the currently available drugs.

## Supplementary Material

We evaluated the activities of A. ha and A. c EO against intracellular amastigotes. These later reside within the macrophages and are responsible for the spread and the severity of the infection in the host. Non-cytotoxic doses of A. ha (68 and 90 µg/ml) and A. c (44 and 60 µg/ml) were assessed for the treatment of mouse peritoneal macrophages infected with L. infantum at a ratio of 1:10 (macrophage: parasites) (Fig. S1). The infection rate decreased of 12 % after the treatment of infected macrophages with A. ha EO at both doses of 68 and 90 µg/ml (Fig. S1 A). While the infection rate in the A. c EO treated-cells decreased by 5-7 % after 24 h treatment (Fig S1 A). The infection rate was 0% within the reference leishmanicidal drug Amphotericin B at 0.25 µg/ml. The time of post-treatment was extended to 72 h in the case of infected peritoneal macrophage with L. major promastigotes at a ratio of 1:15 (macrophage: parasites) (Fig. S2). After treatment of infected cells with both EO at 90 µg/ml A. ha and 60 µg/ml A. c, the infection rate was decreased from 38% to 0% with both EO in comparison with Amphotericin B at 0.25 µg/ml (Fig. S2 A). Giemsa-stained cells observed at 100X magnification showed no remaining amastigotes within the vacuoles (Fig S2 B).

## Figures and Tables

**Table 1:** Chemical composition (%) of *A. campestris *and* A. herba-alba* essential oils.

Compound	RI	*A. campestris*	*A. herba-alba*
*Monoterpene hydrocarbons *			
*α*-Thujene	932	0.41	—
*α*-Pinene	938	12.25	0.26
Camphene	954	0.19	2.86
Sabinene	967	2.35	—
*β*-Pinene	978	32.95	—
*β*-Myrcene	994	5.51	—
*α*-Phellandrene	1007	0.13	—
*α*-Terpinene	1022	1.04	—
p-Cymene	1025	2.28	1.4
*α*-Limonene	1030	15.13	3.99
*α*-Ocimene	1041	3.04	1.99
*β*-Ocimene	1052	3.37	0.61
*γ*-Terpinene	1060	7.60	—
*α*-Terpinolene	1095	0.75	—
*Oxygenated monoterpenes *			
1,8-Cineole	1032	—	13.85
Linalool	1098	0.14	—
Filifolone	1108	—	2.07
*α*-Thujone	1110	—	7.65
*β*-Thujone	1115	—	7.21
Chrysanthenone	1122	—	8.8
Camphor	1147	—	36.82
Pinocarvone	1160	—	1.99
Borneol	1165	—	3.99
Terpinen-4-ol	1176	2.09	—
*α*-Terpineol	1198	0.75	—
Myrtenal	1204	0.18	1.72
Cuminaldhehyde	1236	—	0.87
Bornyl acetate	1286	—	0.82
Geraniol acetate	1390	0.37	—
*Sesquiterpene hydrocarbons *			
*β*-Caryophyllene	1488	0.19	—
Germacrene D	1480	—	0.95
Bicyclogermacrene	1486	0.27	0.61
*Oxygenated sesquiterpenes*			
Spathulenol	1574	0.76	—
Caryophyllene oxide	1585	0.29	—
*β*-Eudesmol	1667	2.91	—

*Total identified (%)*		94.95	98.46

*Monoterpene hydrocarbons (%) *		87	11.11
*Oxygenated monoterpenes (%)*		3.53	85.79
*Sesquiterpene hydrocarbons (%)*		0.46	1.56
*Oxygenated sesquiterpenes (%)*		3.96	—

RI: retention indices relative to *n*-alkanes (C9–C24) on HP-5MS column. —: not detected.

**Table 2:** Antioxidant activities of *A. campestris *and* A. herba-alba* essential oils.

	Free radical scavenging activity (IC_50_; mg/mL)	Ions chelating capacity (mg EDTA/g EO)	Reducing power (mM Fe^2+^)
*A. campestris*	3.3 ± 0.11^b^	2.97 ± 0.03^a^	17.79 ± 0.08^b^
*A. herba-alba*	9.10 ± 0.42^a^	2.30 ± 0.06^a^	27.48 ± 0.30^a^
Trolox	0.025 ± 0.002^c^		

Data are expressed as means ± SD.

In each column, means followed by different letters are significantly different (*p* < 0.05).

## Abbreviations

A. ha: * Artemisia herba-alba*
A. c: * Artemisia campestris*
EO: Essential oil.
