# Supplementary material for: Asteraceae Artemisia campestris and Artemisia herba-alba Essential Oils Trigger Apoptosis and Cell Cycle Arrest in Leishmania infantum Promastigotes
Source: Evid Based Complement Alternat Med. 2016 Oct 11;2016:9147096. doi: 10.1155/2016/9147096 (PMC5078739; doi:10.1155/2016/9147096)
Supplement: Supplementary file 1 — We evaluated the activities of A. ha and A. c EO against intracellular amastigotes. These later reside within the macrophages and are responsible for the spread and the severity of the infection in the host. Non-cytotoxic doses of A. ha (68 and 90 µg/ml) and A. c (44 and 60 µg/ml) were assessed for the treatment of mouse peritoneal macrophages infected with L. infantum at a ratio of 1:10 (macrophage: parasites) (Fig. S1). The infection rate decreased of 12 % after the treatment of infected macrophages with A. ha EO at both doses of 68 and 90 µg/ml (Fig. S1 A). While the infection rate in the A. c EO treated-cells decreased by 5-7 % after 24 h treatment (Fig S1 A). The infection rate was 0% within the reference leishmanicidal drug Amphotericin B at 0.25 µg/ml. The time of post-treatment was extended to 72 h in the case of infected peritoneal macrophage with L. major promastigotes at a ratio of 1:15 (macrophage: parasites) (Fig. S2). After treatment of infected cells with both EO at 90 µg/ml A. ha and 60 µg/ml A. c, the infection rate was decreased from 38% to 0% with both EO in comparison with Amphotericin B at 0.25 µg/ml (Fig. S2 A). Giemsa-stained cells observed at 100X magnification showed no remaining amastigotes within the vacuoles (Fig S2 B). [file 9147096.f1.pdf]

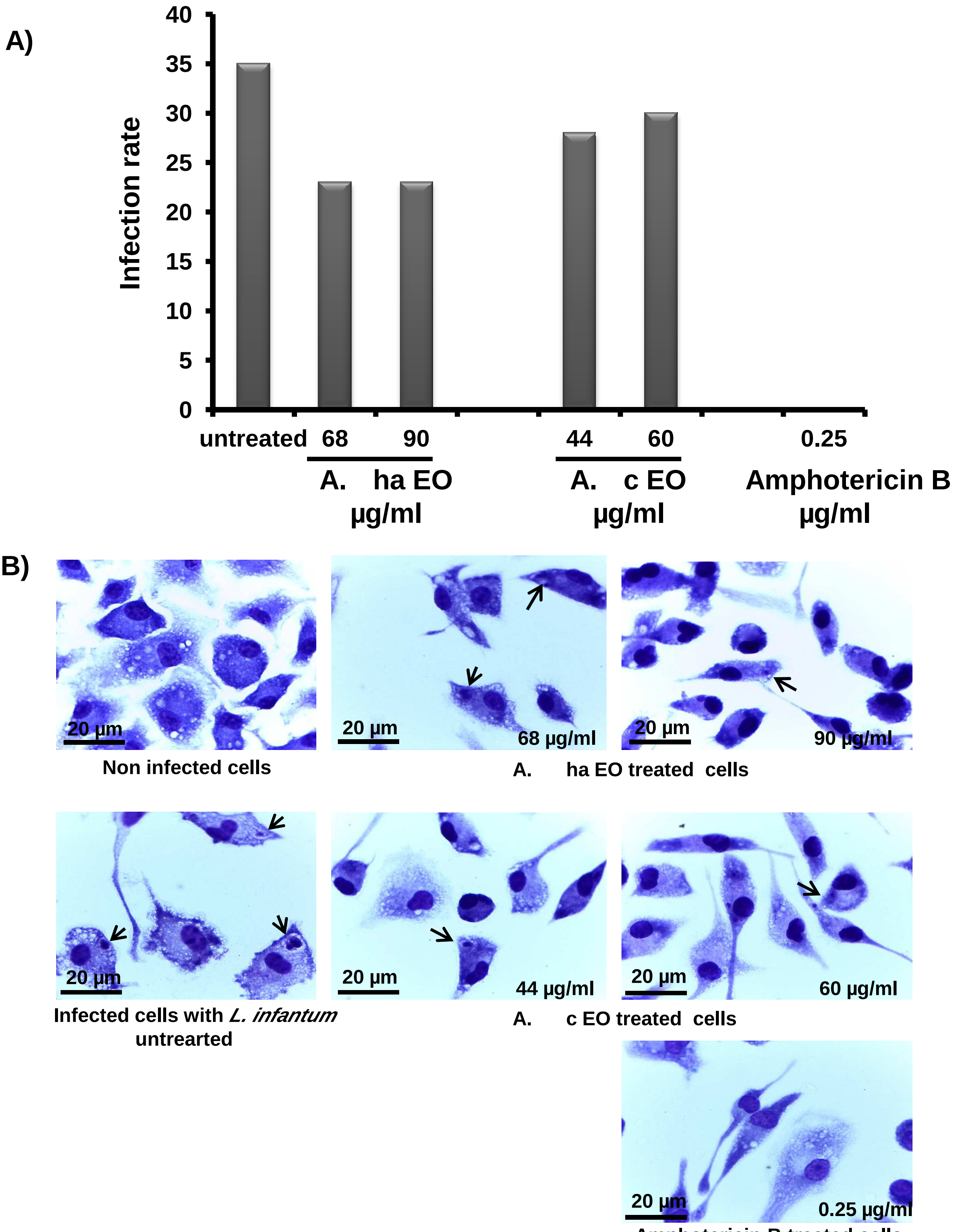

Fig. S1:

A) Effect of A. ha and A. c essential oils on mouse peritoneal macrophages Infected with *L. infantum* at a ratio of 1:10 (macrophage: parasites).

B) Light microscopy of Giemsa-stained cells observed at 100X after 24 h post-treatment. Arrows indicate vacuoles with intracellular amastigotes.

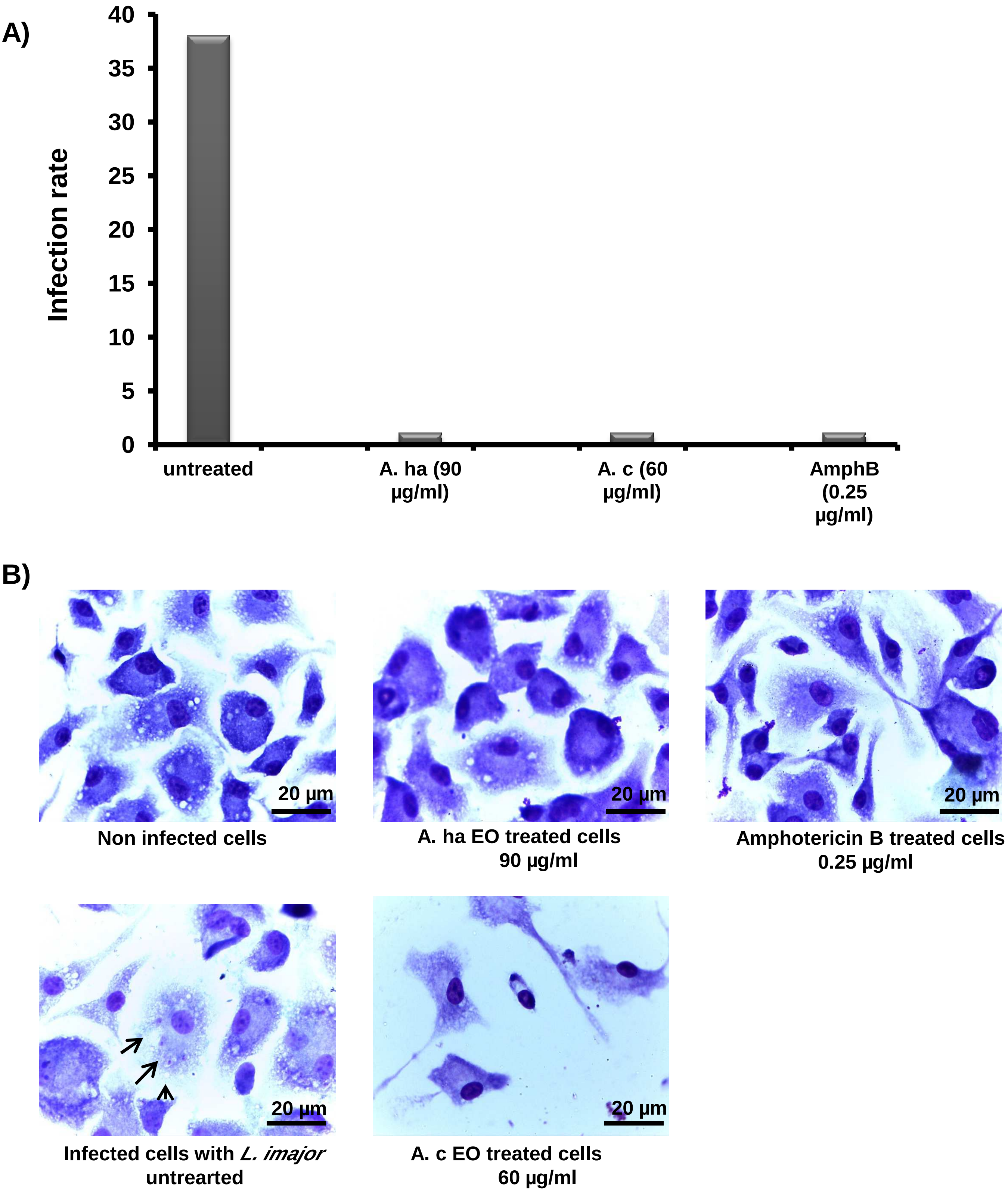

Fig. S2: A) Effect of A. ha and A. c essential oils on mouse peritoneal macrophages Infected with ***L. major*** at a ratio of 1:15 (macrophage: parasites).
B) Light microscopy of Giemsa-stained cells observed at 100 X magnification after 72 h post-treatment. Arrows indicate vacuoles with intracellular amastigotes. No remaining amastigotes in the EO-treated cells.
